# Cerebrospinal Fluid Analysis Should Be Considered in Patients with Cognitive Problems

**DOI:** 10.4061/2010/163065

**Published:** 2010-07-14

**Authors:** Henrik Zetterberg, Niklas Mattsson, Kaj Blennow

**Affiliations:** Department of Psychiatry and Neurochemistry, Institute of Neuroscience and Physiology, The Sahlgrenska Academy at University of Gothenburg, 431 80 Mölndal, Sweden

## Abstract

Hepatologists assay liver enzymes and cardiologists structural heart proteins in serum to diagnose and monitor their patients. This way of thinking has not quite made it into the memory clinics yet, in spite of the availability of validated cerebrospinal fluid biomarkers for key pathological events in the brain in neurodegeneration. Here, we argue that a spinal tap should be considered in all patients who seek medical advice for memory problems and list the highly relevant clinical questions CSF analyses can address.

## 1. Introduction

Memory problems may be caused by a wide range of neuropsychiatric diseases, including Alzheimer's disease (AD), vascular dementia (VaD), dementia with Lewy bodies, frontotemporal dementia (FTD), to mention a few [1]. Cognitive symptoms may also arise secondary to depression, neuroinflammation and various somatic illnesses. Today, patients with memory problems seek medical advice much earlier than 10 years ago. It is difficult to differentiate benign cognitive deficiencies from AD or other primary neurodegenerative diseases. Memory problems secondary to other diseases may also present a diagnostic challenge. 

Patients with memory complaints most often undergo extensive clinical and neuropsychological assessments, and often also one or more brain imaging investigations. We argue that CSF analysis should be considered in the diagnostic work-up of all patients with memory problems to answer a number of highly relevant questions discussed below. Fear of spinal tap-related side-effects should not preclude CSF analyses, since complications are very rare in the elderly, provided that regular precautions well known to any trained physician are taken [2–4].

## 2. Does the Patient Suffer from Brain Amyloid Pathology?

The robust association of brain amyloid pathology with AD makes this question highly relevant. The easiest and most cost-effective way of giving it a reliable answer is to analyse CSF for the 42 amino acid form of amyloid *β* (A*β*1-42). Low CSF levels indicate retention of A*β*1-42 in the brain parenchyma [5–8]. This seems to be the earliest biochemical change during the course of AD [9–11]. Low levels of A*β*1-42 may be seen Creutzfeldt-Jakob disease (CJD), also in the absence of significant amounts of brain amyloid pathology [12].

## 3. Does the Patient Suffer from Neurofibrillary Tangle Pathology?

Tau expression is high in nonmyelinated cortical axons where it serves as a microtubule-stabilizing protein [13]. Hyperphosphorylation of tau causes the protein to detach from the microtubules. This process promotes axonal and synaptic plasticity in the developing brain [14, 15], but is pathological in the adult brain and specifically related to a group of disorders referred to as tauopathies, which includes AD and some forms of FTD [16].

Elevated CSF levels of hyperphosphorylated tau (P-tau) protein are the most specific finding suggesting an ongoing AD process in the brain [17]. P-tau levels correlate with cognitive decline in patients with mild cognitive impairment (MCI) [18] and with neocortical neurofibrillary pathology in AD [19]. The reason for the lack of P-tau increase in FTD and other pure tauopathies such as progressive supranuclear palsy and corticobasal degeneration is to date unknown [20, 21].

## 4. Are there Biochemical Signs of Cortical Axonal Degeneration and How Active Is this Process?

Pathogenic processes that damage axons in the cortex result in increased CSF levels of total tau (T-tau, i.e., all isoforms irrespective of phosphorylation state). T-tau is a dynamic marker of the intensity of the axonal degeneration/damage: the more pronounced increase, the more intense degenerative process, the faster disease progression [17]. Very high CSF T-tau levels are always seen in CJD [22–25], and can be seen in stroke and brain trauma, in which T-tau predicts clinical course and/or outcome [26–28]. The cortical axonal degeneration in AD makes elevated CSF T-tau an obligatory finding. Consequently, a clinical diagnosis of AD should be reconsidered in the absence of tau elevation. Very high levels predict a rapid cognitive decline in AD [29–31] and short survival in DLB [32]. 

Together, CSF biomarkers of amyloid pathology (A*β*1-42), tangle pathology (P-tau) and cortical axonal degeneration (T-tau) identify AD with dementia and prodromal AD in patients with MCI with 75–95% sensitivity and specificity [33–36].

## 5. Are there Biochemical Signs of Sub-Cortical Axonal Degeneration and How Active Is this Process?

The best-established CSF biomarker for sub-cortical axonal degeneration/damage is neurofilament light protein (NFL). This type of axonal degeneration is frequently seen in VaD [37–39], FTD [40] and a number of inflammatory conditions, including MS [41] and AIDS dementia [42]. Elevated CSF NFL levels indicate a sub-cortical disease process and help in differentiating pure AD from the conditions listed above. Combined T-tau and NFL increases indicate mixed forms of AD and cerebrovascular disease.

## 6. Is the Blood-Brain Barrier Damaged?

The best-established biomarker for the integrity of the blood-brain barrier is the ratio of the albumin concentration in CSF to serum (the CSF/serum albumin ratio). Strictly speaking, the CSF/serum albumin ratio is a direct measure of the blood-CSF barrier [43]. However, leaking blood vessels in the brain will eventually result in higher CSF/serum albumin ratio through release of albumin to the brain interstitial fluid which communicates freely with the CSF. Typically, the CSF/serum albumin ratio is normal in patients with pure AD [44], whereas patients with vascular dementia generally present with elevated albumin ratio [45]. The same finding is often present in Lyme disease (neuroborreliosis), where one also may find increased numbers of CSF monocytes and signs of immunoglobulin production within the CNS [46]. Blockage of the spinal canal which, by impairing the flow of CSF distal to the block, allows longer for equilibrium with the circulation and so brings the composition of the CSF nearer to that of plasma (Froin's syndrome), results in elevated CSF/serum albumin ratio in the absence of blood-brain barrier damage.

## 7. Are there Biochemical Signs of Neuroinflammation?

Basic CSF examinations of inflammatory activity, including white blood cell count and general signs of IgG or IgM production within the CNS are generally negative in AD and other primary neurodegenerative diseases [47]. Distinct positive results speak against pure AD and should motivate further investigation of the patient to exclude neuroborreliosis, multiple sclerosis and other neuroinflammatory conditions that may contribute to the cognitive symptoms.

## 8. Conclusions

Robust answers to the clinically relevant questions listed above are warranted in the professional evaluation of any patient with memory complaints. Valid CSF biomarkers are available for amyloid pathology (A*β*1-42), neurofibrillary pathology (P-tau), cortical axonal degeneration/damage (T-tau), sub-cortical axonal degeneration/damage (NFL), blood-brain barrier function (CSF/serum albumin ratio) and neuroinflammation (CSF cell counts, IgG and IgM oligoclonal bands and concentrations). The biochemical data should be interpreted with great care together with the whole clinical picture, as well as findings in neuropsychological testing and neuroimaging investigations. Typical changes in relation to different diagnostic entities are summarised in Table 1.

## Figures and Tables

**Table 1:** CSF biomarkers of pathological findings in relation to differential diagnoses in memory clinic patients.

Diagnosis	Amyloid pathology (A*β*1-42)	Tangle pathology (P-tau)	Cortical axonal damage (T-tau)	Sub-cortical axonal damage (NFL)	Blood-brain barrier dysfunction (CSF/serum albumin ratio)	Inflammation (CSF cell counts, IgG or IgM production)
AD	Yes	Yes	Yes	No	No	No
VaD	No	No	Yes	Yes	Yes	No
			(especially in relation to new brain infarcts)			
FTD	No	Yes	Yes	Yes	No	No
		(but not in CSF)	(mild)			
LBD	Yes	No	Yes	No	No	No
			(mild)			
PD	No	No	No	No	No	No
PSP	No	Yes	No	Yes	No	No
		(but not in CSF)				
CJD	No	No	Yes	Yes	No	No
			(severe)	(mild to moderate)		
Depression	No	No	No	No	No	No
Lyme disease	No	No	No	Yes	Yes	Yes
				(mild)		(especially IgM)
Multiple sclerosis	No	No	No	Yes	No	Yes
					(mild in 10%)	(especially IgG)
Acute stroke	No	No	Yes	Yes	Yes	No
Normal aging	No	No	No	No	No	No

Abbreviations: CSF = cerebrospinal fluid; A*β*1-42 = the 42 amino acid isoform of amyloid *β*; P-tau = hyperphosphorylated tau; T-tau = total tau; NFL = neurofilament light; AD = Alzheimer's disease; VaD = vascular dementia; FTD = frontotemporal dementia; LBD = Lewy body dementia; PD = Parkinson's disease; PSP = progressive supranuclear palsy; CJD = Creutzfeldt-Jakob disease.
